# Age and Clinical Dengue Illness

**DOI:** 10.3201/eid1306.070008

**Published:** 2007-06

**Authors:** Joseph R. Egger, Paul G. Coleman

**Affiliations:** * London School of Hygiene and Tropical Medicine, London, United Kingdom

**Keywords:** dengue fever, relative risk, age, dispatch

## Abstract

The relationship between age and risk for classic dengue fever has never been quantified. We use data from clinical patients to show that the relative risk of having classical disease after primary dengue virus infection increases with age. This relationship has implications for strategies aimed at controlling dengue fever.

Dengue fever has emerged as a serious international public health threat with almost half of the world’s population at risk for infection (*1*). Although >50 million cases of dengue fever are estimated to occur each year (*2*), a large proportion of infections are asymptomatic (*3*).Why infection progresses to clinical disease in some persons, but not in others, is not clear. Some evidence suggests that risk for disease, with both classic dengue fever and the more severe dengue hemorrhagic fever, varies by age (*4*); however, the relationships have never been rigorously quantified. We used data from patients with laboratory-confirmed clinical dengue to describe the relationship between age and the relative risk of becoming ill with classical disease after primary infection with dengue virus. A clearer understanding of this relationship has implications for strategies aimed at controlling dengue fever.

## The Study

Clinical dengue incidence data that have been serologically confirmed were abstracted from a survey conducted in 1997 in the communities of Belém and Ananindeua in Pará State, Brazil (*5*). In 1996, these communities reported their first cases of dengue fever (dengue serotype l) in >50 years, after the successful control of *Aedes aegypti* mosquitoes during the 1940s (*5*). We assumed, therefore, that persons <50 years of age were susceptible to all 4 dengue serotypes at the time of the survey and, as a result, that most reported cases were due to primary dengue infection. Age-stratified population data from the 2000 Brazilian census were used to estimate the total population of Belém and Ananindeua for the following age classes: 0–4, 5–9, 10–14, 15–24, 25–34, 35–44, and 45–54 years. The midpoints of these age classes were used in subsequent statistical analyses. Survey data describing the number of serologically confirmed clinical dengue cases were then used to estimate the minimum proportion of all persons in each age class who had clinical dengue (Figure, Panel A).

**Figure:**
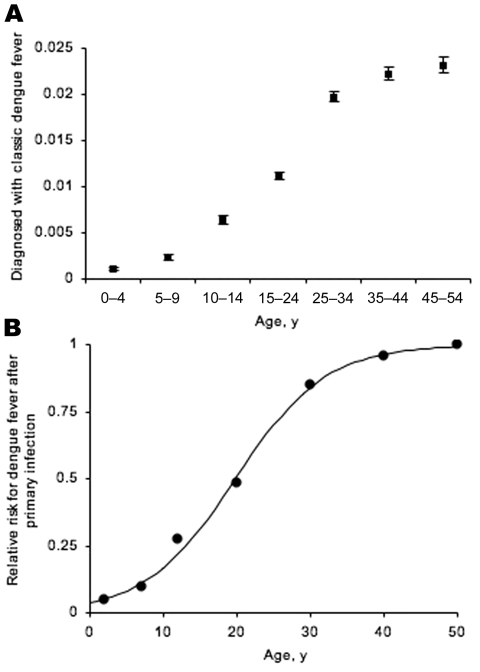
Estimated minimum proportion of the population, by age, with laboratory-confirmed classical dengue, showing exact 95% binomial confidence intervals. A) Fitting a logistic regression model (not shown) to the absolute proportion produced a significant age estimate: McFadden R^2^ = 0.762, χ^2^ = 5,196.13, df = 1, p<0.001. B) Relative risk, by age, of having classical dengue after primary infection. Black circles, observed; line, model fit. See text for details of statistical analysis.

We further investigated the relationship between age and probability of having clinical disease by calculating the risk for each age class relative to the age class that had the highest proportion of clinical cases. Unlike the absolute proportion, the relative risk is independent of transmission intensity. In calculating relative risk, we assumed that the risk for dengue infection was independent of age, which was corroborated by a seroepidemiologic study performed in Fortaleza, Brazil, in 1994 (*6*). A logistic regression model was developed to describe the relationship between age and the relative probability of disease after primary dengue infection. Model fitting was performed with Stata 8.0 (Stata Corporation, College Station, TX, USA), and robust standard errors were calculated for each regression coefficient.

The logistic model provided a significant fit to the data (McFadden R^2^ = 0.512, χ^2^ = 4.86, df = 1, p<0.028) and described a clear positive relationship between age and relative risk for clinical disease (β = 0.164; bootstrap 95% confidence interval 0.1470–0.1769), as shown in the Figure, Panel B. The results suggest that the risk for clinical disease after primary dengue infection is relatively low throughout childhood and then increases rapidly through adolescence and early adulthood.

## Conclusions

To our knowledge, this is the first time data have been used to empirically derive the quantitative relationship between age at time of primary dengue infection and risk of having clinical dengue fever. These findings are consistent with results of earlier studies that suggest that adults are more likely than young children to have clinical dengue (*7*–*9*).

Several factors should be considered when interpreting these results. First, because dengue virus serotypes l and 2 were circulating in the population during the study period, some persons may have been infected with both serotypes during the 1-year period and, therefore, clinical signs may have resulted from a secondary infection. This proportion is probably small. Second, several factors other than age are thought to influence severity of classic dengue illness, including viral serotype and strain (*4*,*10*). Data from a dengue epidemic (dengue virus type 3) in Puerto Rico showed the attack rate to be independent of age (*11*). Although the proportion of these cases that were due to primary infection was uncertain, the different infecting serotype may be partly responsible for the conflicting findings between that study and ours. Further research should be conducted to determine whether the relationship between age and classic dengue fever is similar in epidemics involving all 4 dengue virus serotypes. Finally, whether all age groups in the study population had equal access to participating health facilities is not known. However, if a reporting bias were introduced, it would likely be in adults (because of child-rearing duties and difficulty taking time off work). Therefore, because adults represent a higher proportion of total patients with clinical cases in this study, underreporting in this age group would suggest that our relative-risk estimates in the adult age classes are conservative.

Despite the complexities of dengue epidemiology, these findings provide strong empirical evidence that age is an important factor in determining risk for disease severity after primary dengue virus infection. As such, these findings have important implications for initiatives aimed at controlling dengue. Interventions focused on reducing the number of *Aedes* mosquitoes are the mainstay of dengue control worldwide. Such approaches, however, have proved incapable of interrupting dengue transmission (*12*). At best, vector control may result in a partial reduction in the rate at which dengue virus is transmitted, which consequently increases the average age of the population susceptible to dengue infection. If age is a risk factor for clinical dengue fever, as our results suggest, then while partial control will decrease the rate of dengue infection, it may have the adverse effect of increasing clinical incidence.
